# Predictive Factors of Central-Compartment Lymph Node Metastasis for Clinical N0 Papillary Thyroid Carcinoma With Strap Muscle Invasion

**DOI:** 10.3389/fendo.2020.00511

**Published:** 2020-09-08

**Authors:** Shuai Xue, Li Zhang, Renzhu Pang, Peisong Wang, Meishan Jin, Liang Guo, Yuhua Zhou, Bingfei Dong, Guang Chen

**Affiliations:** ^1^Department of Thyroid Surgery, The First Hospital of Jilin University, Changchun, China; ^2^Department of Nephrology, The First Hospital of Jilin University, Changchun, China; ^3^Department of Pathology, The First Hospital of Jilin University, Changchun, China

**Keywords:** predictive factors, central-compartment lymph node metastasis, clinical N0, papillary thyroid carcinoma, strap muscle invasion

## Abstract

**Background:** Papillary thyroid carcinoma (PTC) patients with anterior extrathyroidal extension (ETE) involving the strap muscle have a relatively better prognosis than those with posterior gross ETE involving the recurrent laryngeal nerve. Whether prophylactic central-compartment lymph node dissection (CLND) should be performed in PTCs with only strap muscle invasion (SMI) is still unclear.

**Methods:** A retrospective cohort study was conducted in clinical N0 (cN0) PTC patients with SMI who underwent thyroid surgery from 2009 to 2017. A total of 152 patients were included, and predictive factors of central-compartment lymph node metastasis (CLNM) were determined.

**Results:** Among the 281 PTCs patients with SMI, 152 (51.1%) did not clinically present with lymph node metastasis. Microscopic CLNM was identified in 77 (50.7%) cN0 PTC patients with SMI. According to the univariate and multivariate analyses, male patients and those aged <40 years were more likely to be diagnosed with CLNM than female patients and those aged >40 years (odds ratio [OR] = 6.22 [95% confidence interval (CI), 1.43–27.10], *p* = 0.02 vs. OR = 9.94 [95% CI, 2.79–35.44], *p* = 0.00). The CLNM positive rate of male patients aged <40 years was 87.5%, while that for female patients aged ≥55 years was 23.8%. However, risk factors associated with large-volume CLNM were not identified because of the small number of patients.

**Conclusions:** Taken together, nearly half of PTC patients with SMI did not clinically present with lymph node metastasis. Male sex and patients aged <40 years were identified as the predictive factors of CLNM in cN0 PTCs with SMI. Hence, the results of this single-center study raise the possibility that prophylactic CLND may be more often considered for younger male PTC patients with SMI.

## Introduction

The incidence of papillary thyroid carcinoma (PTC) has significantly increased worldwide during the past decades (1, 2). Central-compartment lymph node metastasis (CLNM), considered a poor clinical feature, is associated with the prognosis of PTC (3, 4). Patients with >5 lymph node metastasis (LNM), are associated with structural recurrence, distant metastasis, and mortality (4, 5), even despite being micrometastases. Therefore, prophylactic central-compartment lymph node dissection (CLND) was recommended for T3 or T4 primary tumors, or if the information of LNM would be used to plan further treatment strategies, like completion thyroidectomy or radioiodine ablation (5). However, the sensitivity and specificity of ultrasonography (US) for diagnosing CLNM is poor (6, 7). Therefore, several preoperative clinical factors of CLNM for PTC have been identified by several studies, suggesting that high-risk PTCs require aggressive treatment (8, 9). However, the conflicting results from these studies contribute to different therapeutic strategies for PTC (10).

Extrathyroidal extension (ETE) is defined as a tumor spread outside of the thyroid and into the surrounding tissues (11). The degree of extrathyroidal extension (ETE) in PTC plays a significant role in recurrence and mortality (11). The American Thyroid Association guidelines recommend CLND in PTCs with gross ETE (5). Along with the degree of gross ETE, the site of the tumor gross invasion is also associated with disease outcome. However, only strap muscle invasion (SMI) has an effect on the prognosis of PTC, which has already been confirmed by a substantial amount of evidence (12–15). It was reported that DTC patients with only gross SMI had the same recurrent rate as those with microscopic ETE (12–14). Patients with anterior SMI have a relatively better prognosis compared with patients with posterior gross ETE involving the recurrent laryngeal nerve or esophagus because SMI can be easily resected with negative margins (16, 17). Hence, whether prophylactic CLND should only be performed in PTCs with only SMI is still unclear.

This study aimed to evaluate CLNM in clinical N0 (cN0) PTC with only SMI by performing a retrospective analysis of our clinical PTC cohort and identifying the clinicopathological features to predict CLNM, which may guide physicians in planning further treatment strategies.

## Materials and Methods

### Patient Selection

**Figure 1:**
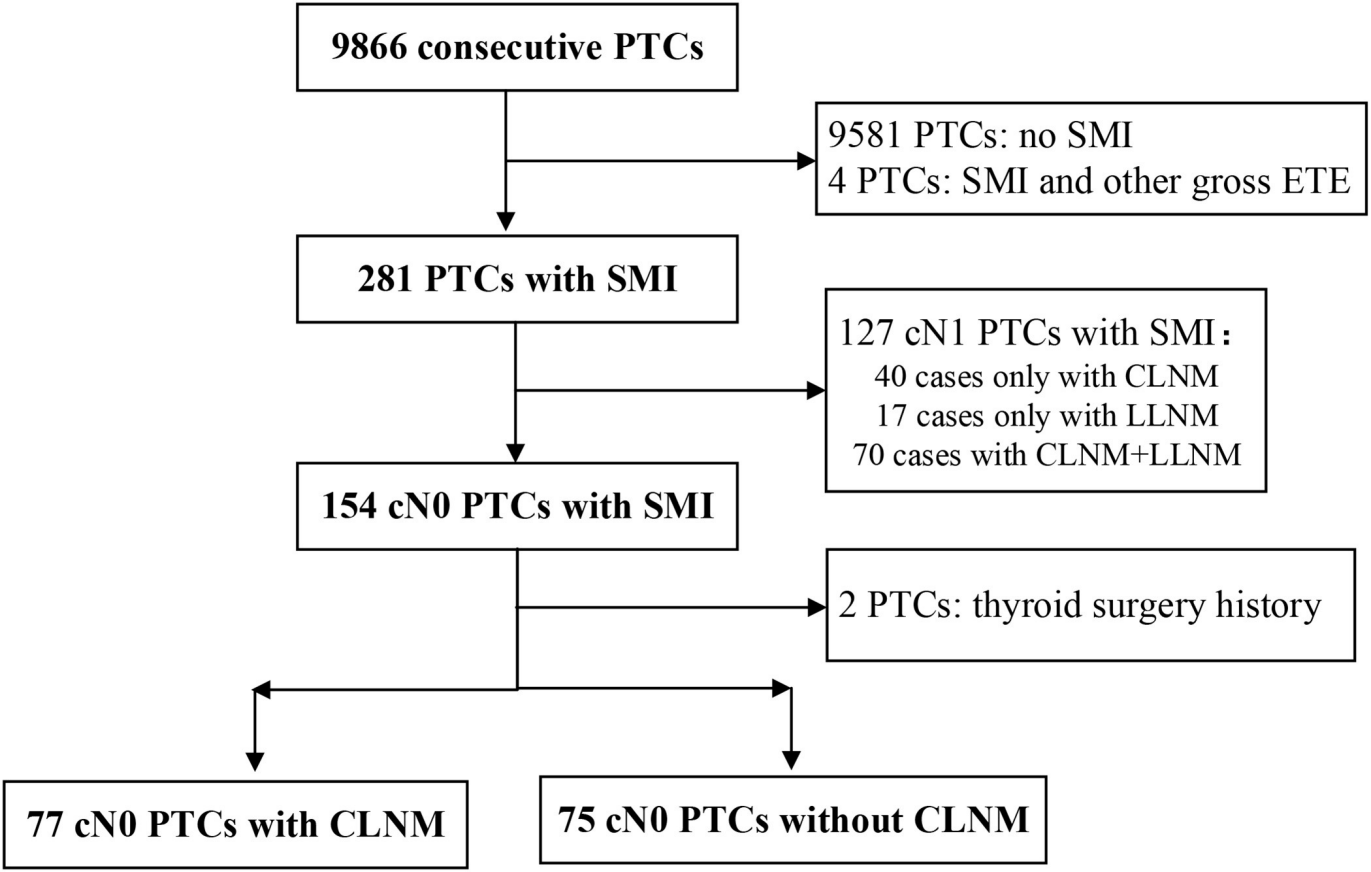
Flowchart of patient selection.

This retrospective study was approved by the Institutional Review Board of the First Hospital of Jilin University, and the need for informed consent was waived. A total of 9,866 consecutive PTC patients from January 2009 to July 2017 who underwent surgery at our department were analyzed retrospectively. The inclusion criteria for patient selection were as follows: (1) patient information found in a hospital database; (2) postoperative pathological diagnosis of conventional PTC with SMI (without other gross ETEs); and (3) absence of suspicious cervical lymph nodes observed during US, computed tomography (CT), and/or fine-needle aspiration (FNA) preoperatively (cN0). The exclusion criteria were as follows: (1): age <18 years, (2) history of neck radiotherapy, and (3) history of previous thyroid surgery. Finally, 152 PTC patients with SMI were enrolled in our study. The flowchart of patient selection is shown in Figure 1.

### Diagnosis and Treatment

The majority of PTCs were identified by US examination, which was performed to evaluate thyroid tumor and neck lymph nodes by a trained radiologist (Y Yin) and surgeons preoperatively (SX, RP, and J Liu). FNA was recommended in patients with suspicious thyroid nodules ≥5 mm. For suspicious thyroid nodules <5 mm, after providing a full explanation of the potential risks and benefits of surgery, patients' decision on whether to undergo surgery was considered. Suspicious SMI was previously defined by US according to the following criteria: (1) a tumor was located in the anterior portion of the thyroid and (2) the thyroid capsule was disrupted by the growing tumor, or >25% of the tumor perimeter was abutting the thyroid capsule. All patients with suspicious SMI underwent neck-enhanced CT to further evaluate the SMI and cervical lymph nodes. FNA for suspicious lymph nodes was recommended when the largest diameter of the cervical lymph node was >0.8 cm and when patients presented with ≥1 malignant US/CT features (microcalcifications, cystic aspect, peripheral vascularity, hyperechogenicity, and rounded shape). The total thyroid and invaded strap muscle were dissected. Bilateral prophylactic CLND was performed (18). Radioactive iodine and thyroid-stimulating hormone-suppressive hormonal therapy were recommended to postoperative patients according to the established guidelines (5).

### Histopathological Examination

Histological specimens were examined and reviewed (19). Histopathological characteristics, including the largest tumor diameter (LTD), location of the tumor, ETE, presence of Hashimoto thyroiditis (HT), and LNM (the number and diameter), were recorded. The concordance rate between the two pathologists for the 152 enrolled patients in this study was 100%.

### Statistical Analysis

Nominal variables were described as a frequency with a percentage and a mean with a standard deviation for continuous variables. To identify the differences between groups for specific variables, the Statistical Package for the Social Sciences (SPSS) version 22 software (SPSS Inc., Chicago, IL) was used for statistical analysis. Nominal variables and continuous variables were assessed by performing Pearson's chi-squared test and Mann-Whitney *U*-test. A logistic regression model was used to evaluate the risk factors for CLNM and high-volume CLNM (≥5-mm metastatic central lymph nodes). A *p* < 0.05 was considered statistically significant (two-sided).

## Results

### Baseline Characteristics

Among the 281 PTC patients with SMI, 152 (51.1%) did not clinically present with lymph node metastasis. The baseline clinicopathological and genetic characteristics of the 152 PTC patients with SMI are summarized in Table 1. A total of 136 (89.5%) patients were female, and the average age of all patients was 46.9 years. Ninety-five (62.5%) patients presented with a solitary tumor, while a total of 57 (37.5%) patients presented with multifocal tumors. Microscopic CLNM was identified in 77 (50.7%) cN0 PTC patients with SMI.

**Table 1:** Clinicopathological characteristics of cN0 PTC with SMI.

**Variables**	***N* = 152 (%)**
**Sex**	
Female	136 (89.5)
Male	16 (10.5)
**Age, Years**	46.9 ± 9.5
<40	36 (23.7)
40–54	92 (60.5)
≥55	24 (15.8)
**Bilateral**	
Yes	65 (42.8)
No	87 (57.2)
**Location of tumor**	
Solitary tumor	95 (62.5)
Upper third	29 (30.5)
Middle third	37 (38.9)
Lower third	29 (30.5)
Multifocal tumor	57 (37.5)
In both lobes	42 (73.7)
In one lobe	15 (26.3)
**LTD (cm)**	1.4 ± 0.7
**HT**	
Yes	55 (36.2)
No	97 (63.8)
**CLNM**	
Yes	77 (50.7)
No	75 (49.3)
**Metastatic CLN**	3.12 ± 2.01
**Removed CLN**	7.33 ± 4.45

*Categorical variables are presented as number (%, percentage). Continuous variables are presented as the average ± standard deviation. PTC, papillary thyroid carcinoma; LTD, largest tumor diameter; HT, Hashimoto's thyroiditis; CLNM, central lymph node metastasis; CLN, central lymph node*.

### Univariate and Multivariate Analysis of Risk Factors for CLNM

**Figure 2:**
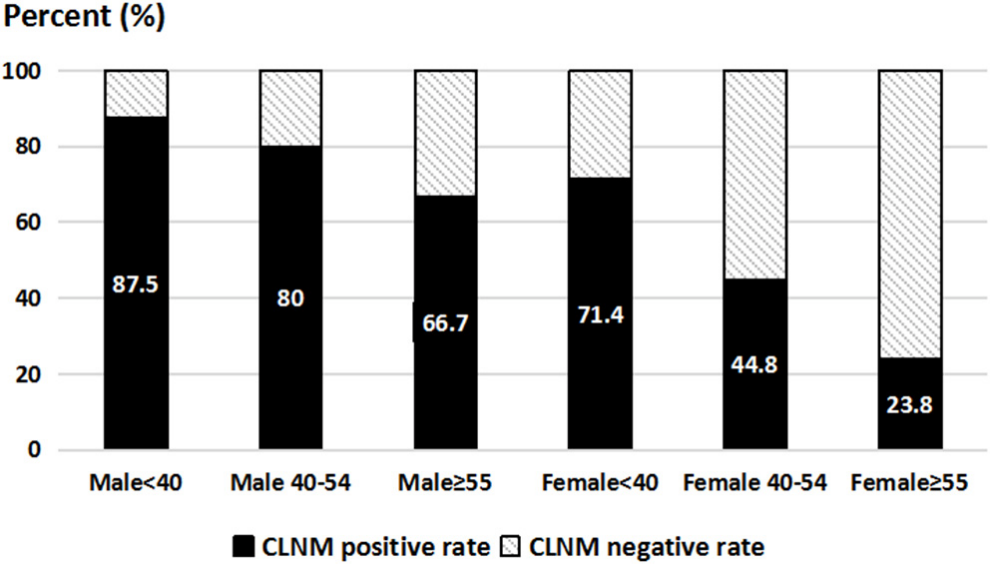
Central-compartment lymph node metastasis rate according to sex and age.

To identify the risk factors for CLNM in cN0 PTCs with SMI, univariate analysis for variables associated with CLNM was performed, including sex, age, bilaterality, multifocality, location of the tumor, LTD, and HT. According to the univariate and multivariate analyses, males and patients aged <40 years were more likely to be diagnosed with CLNM (odds ratio [OR] = 6.22 [95% confidence interval (CI), 1.43–27.10], *p* = 0.02; OR = 9.94 [95% CI, 2.79–35.44], *p* = 0.00) than females and patients aged >40 years, as shown in Table 2. The CLNM positive rate of male patients aged <40 years was 87.5%, while it was only 23.8% in female patients aged ≥55 years, as indicated in Figure 2.

**Table 2:** Univariate and multivariate analysis of clinicopathological characteristics for CLNM in cN0 PTCs with SMI.

**Variables**	**CLNM (+)**	**CLNM (-)**	**Univariate analysis**		**Multivariate analysis**	
	***n* = 77,%**	***n* = 75,%**	**Odd ratio (95% CI)**	***P*-value**	**Odd ratio (95% CI)**	***P*-value**
**Sex**						
Female	64 (83.1)	72 (96.0)	1 (reference)		1 (reference)	
Male	13 (16.9)	3 (4.0)	4.88 (1.33–17.88)	0.02	6.22 (1.43–27.10)	0.02
**Age, Years**						
<40	27 (35.1)	9 (12.0)	7.29 (2.29–23.22)	0.00	9.94 (2.79–35.44)	0.00
40–54	43 (55.8)	49 (65.3)	2.13 (0.81–5.63)	0.13	2.65 (0.92–7.65)	0.07
≥55	7 (9.1)	17 (22.7)	1 (reference)		1 (reference)	
**Bilateral**						
Yes	36 (46.8)	29 (38.7)	1.39 (0.73–2.66)		1.51 (0.65–3.52)	
No	41 (53.2)	46 (61.3)	1 (reference)	0.31	1 (reference)	0.34
**Multifocality**						
Yes	32 (41.6)	25 (33.3)	1.42 (0.73–2.75)		1.72 (0.71–4.18)	
No	45 (58.4)	50 (66.7)	1 (reference)	0.30	1 (reference)	0.23
**Location of tumor with SMI**						
Upper third	17 (22.1)	22 (29.3)	1 (reference)		1 (reference)	
Middle third	33 (42.9)	30 (40.0)	1.42 (0.64–3.18)	0.39	1.31 (0.52–3.26)	0.57
Lower third	27 (35.0)	23 (30.7)	1.52 (0.65–3.53)	0.33	1.87 (0.73–4.76)	0.19
**LTD (mm)**						
≤ 10	22 (28.6)	27 (36.0)	1 (reference)		1 (reference)	
>10	55 (71.4)	48 (64.0)	1.41 (0.71–2.78)	0.33	2.03 (0.92–4.49)	0.08
**HT**						
Yes	30 (39.0)	25 (33.3)	1.28 (0.66–2.48)		1.44 (0.68–3.02)	
No	47 (61.0)	50 (66.7)	1 (reference)	0.47	1 (reference)	0.34

*CLNM, central lymph node metastasis; PTC, papillary thyroid carcinoma; SMI, strap muscle invasion; LTD, largest tumor diameter; HT, Hashimoto's thyroiditis*.

### Univariate and Multivariate Analysis of Risk Factors for Large-Volume CLNM

To identify the risk factors for large-volume CLNM in cN0 PTCs with SMI, univariate analysis for variables associated with large-volume CLNM was performed, including sex, age, bilaterality, multifocality, location of the tumor, LTD, and HT. According to the univariate and multivariate analyses, risk factors associated with large-volume CLNM were not identified, as shown in Table 3.

**Table 3:** Univariate and multivariate analysis of clinicopathological characteristics for large volume CLNM in cN0 PTCs with SMI.

**Variables**	**Large volume CLNM**	**Small volume CLNM**	**Univariate analysis**		**Multivariate analysis**	
	***n* = 18,%**	***n* =134,%**	**Odd ratio (95% CI)**	***P*-value**	**Odd ratio (95% CI)**	***P*-value**
**Sex**						
Female	14 (77.8)	122 (91.0)	1 (reference)		1 (reference)	
Male	4 (22.2)	12 (9.0)	2.90 (0.82–10.24)	0.10	2.30 (0.59–8.93)	0.23
**Age, Years**						
<40	7 (38.9)	29 (21.6)	0.67 (0.16–2.73)	0.57	0.63 (0.15–2.76)	0.54
40–54	8 (44.4)	84 (62.7)	1.69 (0.39–7.31)	0.48	1.57 (0.34–7.18)	0.56
≥55	3 (16.7)	21 (15.7)	1 (reference)		1 (reference)	
**Bilateral**						
Yes	11 (61.1)	57 (42.5)	1.08 (0.40–2.91)		1.94 (0.56–6.72)	
No	7 (38.9)	77 (57.5)	1 (reference)	0.88	1 (reference)	0.30
**Multifocality**						
Yes	6 (33.3)	51 (38.1)	0.81 (0.29–2.30)		0.67 (0.19–2.39)	
No	12 (66.7)	83 (61.9)	1 (reference)	0.70	1 (reference)	0.54
**Location of tumor with SMI**						
Upper third	4 (22.2)	35 (26.1)	1 (reference)		1 (reference)	
Middle third	8 (44.4)	55 (41.1)	1.27 (0.36–4.55)	0.71	1.30 (0.33–5.05)	0.71
Lower third	6 (33.3)	44 (32.8)	1.19 (0.31–4.56)	0.80	1.23 (0.31–5.00)	0.77
**LTD (mm)**						
≤ 10	7 (38.9)	42 (31.3)	1 (reference)		1 (reference)	
>10	11 (61.1)	92 (68.7)	0.72 (0.26–1.98)	0.52	0.70 (0.23–2.11)	0.53
**HT**						
Yes	5 (27.8)	50 (37.3)	0.65 (0.22–1.92)		0.65 (0.21–2.00)	
No	13 (72.2)	84 (62.7)	1 (reference)	0.43	1 (reference)	0.45

*CLNM, central lymph node metastasis; PTC, papillary thyroid carcinoma; SMI, strap muscle invasion; LTD, largest tumor diameter; HT, Hashimoto's thyroiditis*.

## Discussion

To the best of our knowledge, this is the first paper to determine the risk factors for CLNM in cN0 PTCs with SMI. In our 152 PTC patients with SMI, only 77 of the 152 patients (50.7%) presented with microscopic CLNM. Moreover, only 18 patients (11.8%) were reported to have large-volume CLNM (≥5-mm metastatic central lymph nodes). Furthermore, male sex and patients aged <40 years were identified as the risk factors for CLNM in cN0 PTCs with SMI.

It has been well-known that prognosis is worse in patients with gross ETE than in those with microscopic local invasion (11). However, SMI has little effect on the outcome of PTC patients, which has already been confirmed by a substantial amount of evidence (12, 16). Moran et al. reported that non-metastatic differentiated thyroid cancer patients with SMI shared similar locoregional recurrence-free rates with those with microscopic ETE (20). Compared with patients with gross ETE in the trachea, esophagus, and recurrent laryngeal nerve, patients with anterior SMI had a relatively better outcome because the invaded strap muscle can be resected easily with negative margins (17, 21, 22). Accordingly, some researchers recommended that the actual effects of SMI should be reevaluated and revised in future staging systems.

Whether routine prophylactic CLND should only be performed in PTCs with only SMI has remained controversial. The potential benefits of performing routine prophylactic CLND, such as better risk stratification of recurrence according to micrometastatic central lymph nodes and lower thyroglobulin levels after operation, should be balanced by the potential risks, such as permanent hypoparathyroidism (23, 24). Hence, a number of researchers believed that prophylactic CLND was an optimal treatment for cN0 PTCs with risk factors (25). Additionally, we previously summarized a total of 1,555 cN0 PTC patients and identified that male sex and younger age were considered as the risk factors for CLNM (25). Andrew MT also reported that younger age and male sex were the strongest predictive factors for CLNM in PTC cases, which is consistent with our results (26). Regarding papillary thyroid microcarcinoma (PTMC), multiple studies have also found that male sex and younger age were associated with CLNM (27, 28). Moreover, large-volume CLNM was more frequently observed in younger and male PTMC patients than in older and female PTMC patients (29). Considering the significant number of debates on prophylactic CLND, it is recommended only in PTC patients with some risk factors. In our study, only 50.7% of cN0 PTC patients with SMI presented with microscopic CLNM. Furthermore, the CLNM positive rate of male patients aged <40 years was 87.5%. Accordingly, prophylactic CLND may be more often considered for younger male PTC patients with SMI.

This study has several limitations. First, this study was a retrospective single-center study, which may limit the generalization of the findings on a broader scale because of selection bias. Hence, prospective studies with a randomized controlled selection might be required. Second, considering the small number of cN0 PTC with SMI, predictive factors may be generated using a multivariate logistic regression model with some biases. Finally, the difference of disease outcome between SMI patients with and without CLNM was not reported in our study because of the lack of follow-up data. Despite these limitations, our study was the first study to analyze the risk factors of CLNM in cN0 PTC with SMI. Furthermore, sex and age can be easily identified preoperatively. It may have potential significant implications for prophylactic CLND in cN0 PTCs with SMI.

## Conclusion

In conclusion, nearly half of PTC patients with SMI did not clinically present with lymph node metastasis. Male sex and patients aged <40 years were identified as the predictive factors of CLNM in cN0 PTCs with SMI. The results of this single-center study suggest the possibility that prophylactic CLND may be more often considered for younger male PTC patients with SMI.

## Data Availability Statement

The datasets analyzed in this article are not publicly available. Requests to access the datasets should be directed to Guang Chen, jidayiyuanjzx@sina.com.

## Ethics Statement

The studies involving human participants were reviewed and approved by The Institutional Review Board of the 1st Hospital of the Jilin University. Written informed consent for participation was not required for this study in accordance with the national legislation and the institutional requirements.

## Author Contributions

All authors listed have made a substantial, direct and intellectual contribution to the work, and approved it for publication.

## Conflict of Interest

The authors declare that the research was conducted in the absence of any commercial or financial relationships that could be construed as a potential conflict of interest.
